# Supplementary material for: DNA-assisted swarm control in a biomolecular motor system
Source: Nat Commun. 2018 Jan 31;9:453. doi: 10.1038/s41467-017-02778-5 (PMC5792447; doi:10.1038/s41467-017-02778-5)
Supplement: Supplementary file 1 — Supplementary Information [file 41467_2017_2778_MOESM1_ESM.pdf]

# DNA-assisted swarm control in a biomolecular motor system

Keya et al.

## Supplementary Methods

### Measurement of the labeling ratio of *r*-DNAs to tubulin dimers

The *r*-DNA conjugated MTs were depolymerized to *r*-DNA conjugated tubulins by keeping the MTs on ice overnight. The absorbance spectrum of the *r*-DNA conjugated tubulin dimers was measured using a spectrophotometer (NanoDrop™ 2000c, Thermo Fisher Scientific Inc.) and deconvoluted using the normal distribution function with Microsoft Excel 2016 (Windows Edition, Microsoft Corporation) with peaks at 260 nm and 280 nm. The concentrations of DNA and tubulin dimers were calculated from the Beer-Lambert law using molar extinction coefficient of tubulin dimers (115,000 L $mol^{-1}$ $cm^{-1}$), *r*-DNA1 (130,200 L $mol^{-1}$ $cm^{-1}$) and *r*-DNA2 (134,700 L $mol^{-1}$ $cm^{-1}$), from which the labeling ratio was determined (Supplementary Fig. 1 and Supplementary Tables 1 and 2).

### Measurement of the melting temperature ($T_m$) of the *p*-DNA duplex

The *p*-DNA duplex (2 µM) was dissolved in 10 mM phosphate buffer (pH 7.0) with 100 mM NaCl. The *p*-DNA duplex in the *cis* and *trans* state were obtained by irradiating with a xenon light source (MAX-301, Asahi Spectra, Tokyo, Japan) equipped with interference filters at 340 nm (half bandwidth 9 nm) and 450 nm (half bandwidth 9 nm). Before the $T_m$ measurement, either 340 nm or 450 nm light was irradiated to the *p*-DNA duplex solution for 3 min. The melting curves of the duplex in the *cis* and *trans* state were obtained with a spectrophotometer (UV-1800; Shimadzu Inc. Japan) at 260 nm in a quartz cell with 10 mm path length[1] (Supplementary Fig. 5). The heating rate was 0.5 °C/min. $T_m$ was determined from the maximum in the first derivative of the melting curve.

### Image analysis and measurement of the association ratio of MTs

The length and velocity of MTs were measured from images and movies of motility assays captured by fluorescence microscopy using image analysis software (ImageJ).

The association ratio at a given time *t* was determined by counting the number of single MTs manually and dividing the number at time *t* by the number present initially ($t$ = 0). The time-dependent association ratio *R(t)* of red and green MTs was determined as follows

$$R(t) = \frac{N(0) - N(t)}{N(0)} \qquad \text{.................................. (1)}$$

with

$N(0)$ = Initial number of single MTs,

$N(t)$ = Number of single MTs after time $t$.

The mean association ratio was obtained from the average of four regions of interest (126.5 μm ×126.5 μm).

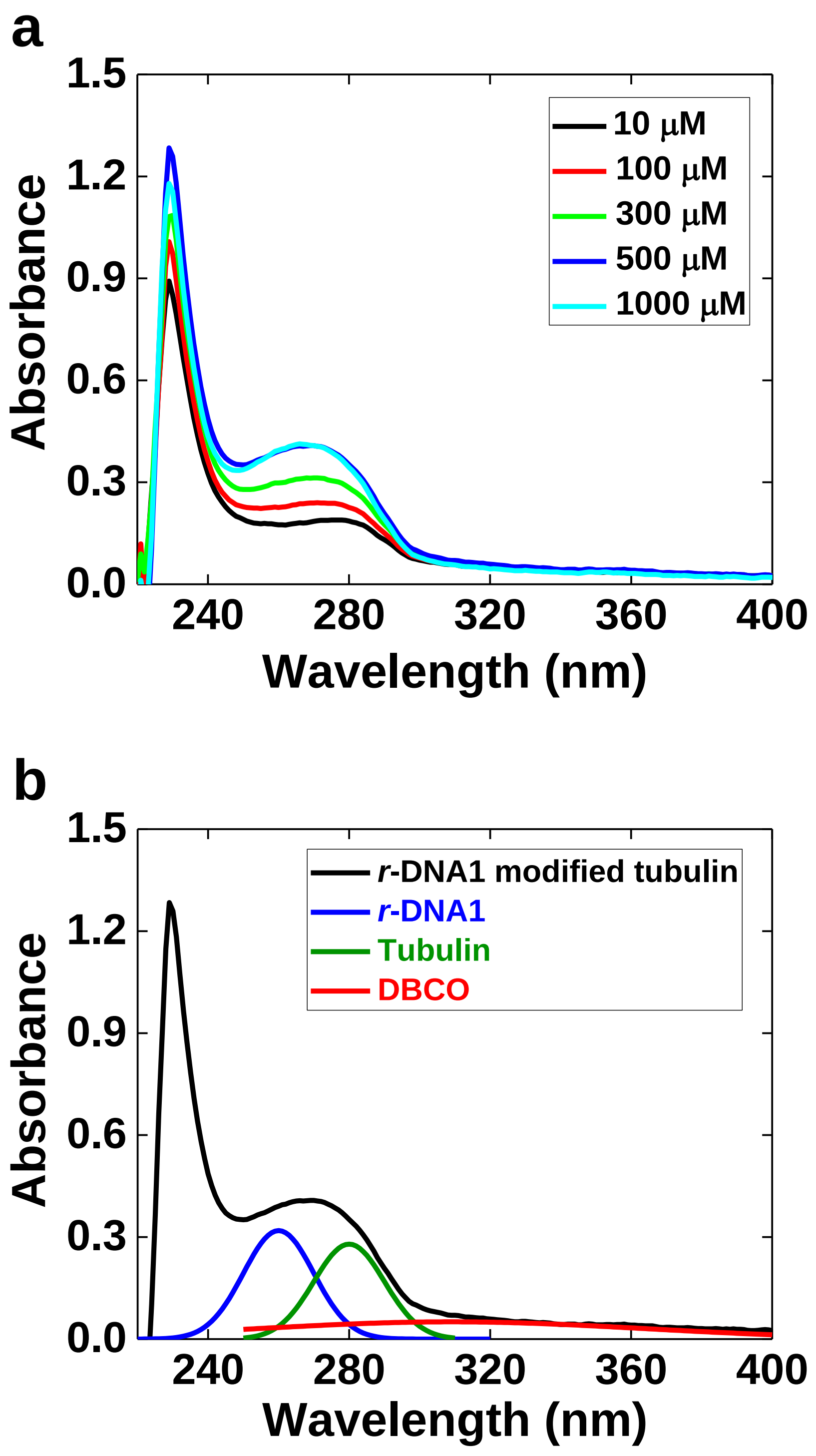


**Supplementary Figure 1. Evaluation of the labeling ratio of *r*-DNA1 after conjugation to rigid MTs (GMPCPP-MTs). a,** Representative absorption spectra of *r*-DNA1 conjugated MTs. The legend shows the concentration of *r*-DNA1 in the feed used to conjugate it to the MTs. **b,** Representative deconvoluted absorption spectra for *r*-DNA1 (500 μM), tubulin and DBCO.

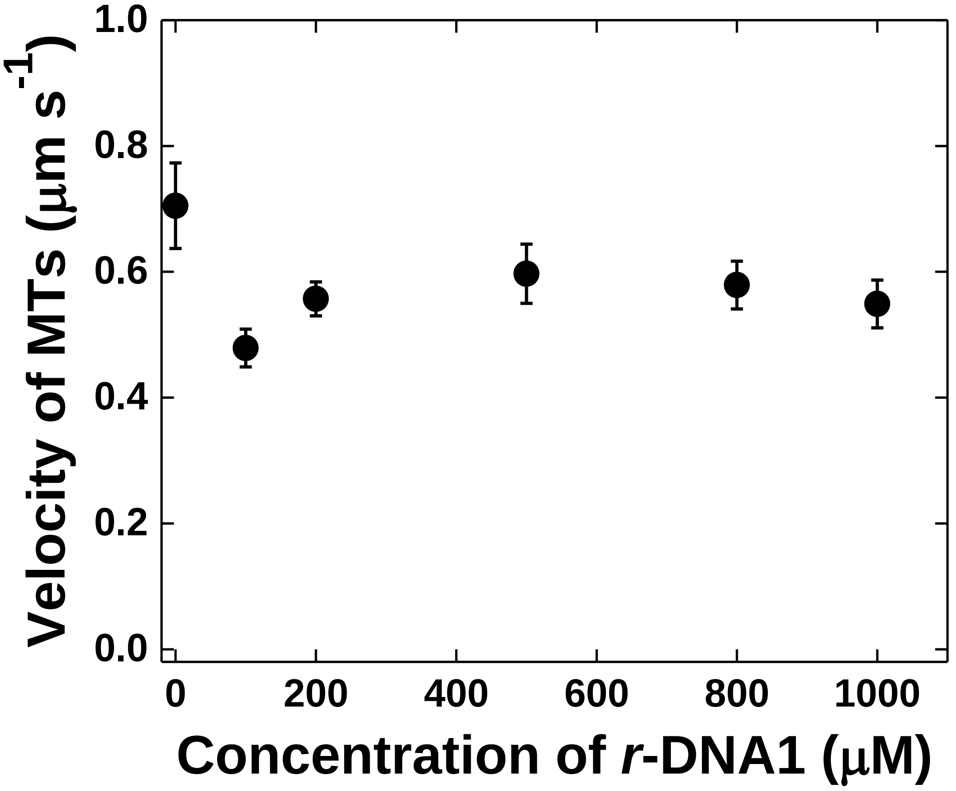


**Supplementary Figure 2. Effect of the concentration of *r*-DNA1 in the feed on the velocity of the rigid MTs (GMPCPP-MTs).** The velocity was measured at 25 °C and the number of MTs considered was 30. The kinesin concentration was 0.3 μM. Error bar: Standard Error (s.e.m.).

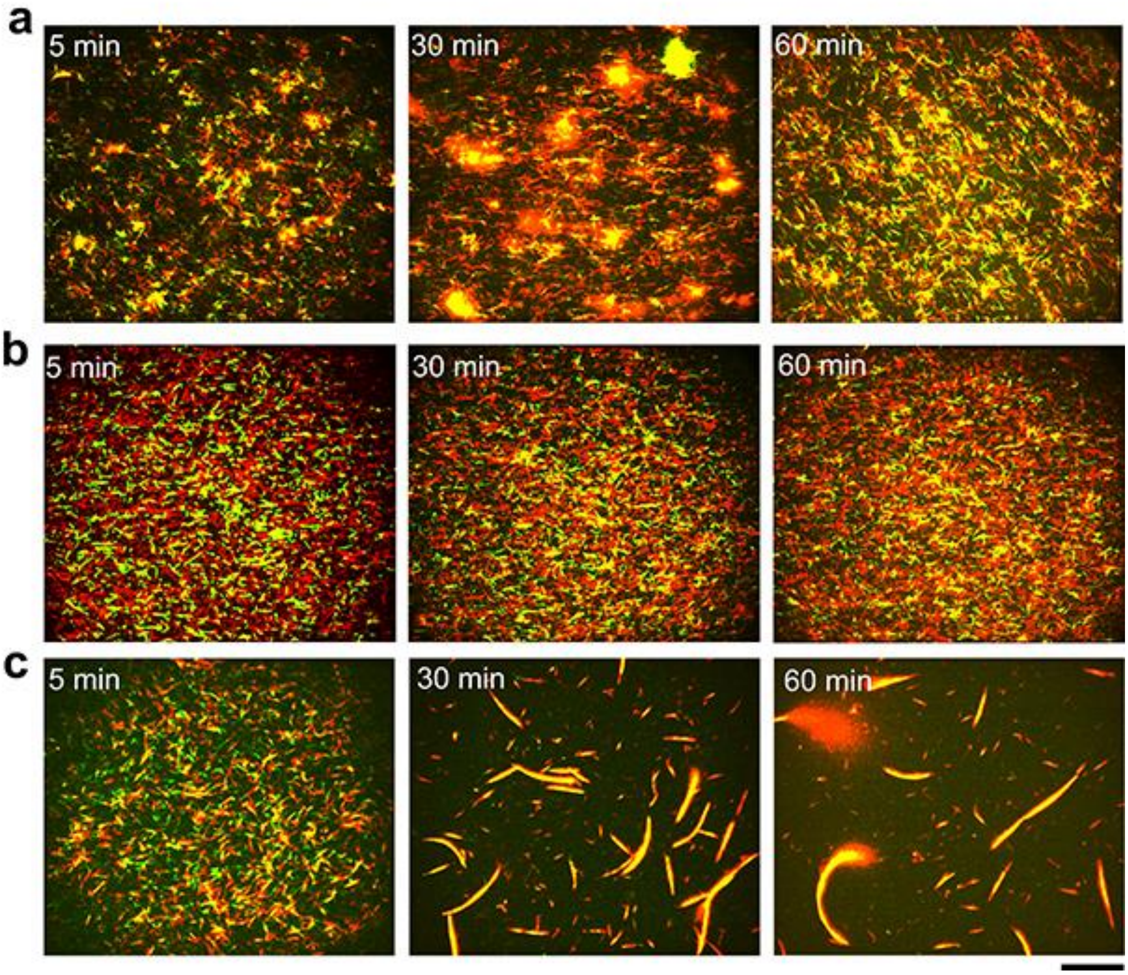


**Supplementary Figure 3. Role of kinesin and ATP on the swarming of *r*-DNA1 and *r*-DNA2 conjugated rigid MTs (GMPCPP-MTs). a,** Fluorescence microscopy images showing the unstructured aggregates of the MTs formed in the absence of kinesin. **b,** Fluorescence microscopy images showing the immobilized MTs on a kinesin coated surface in the absence of ATP. **c,** Fluorescence microscopy images showing the swarms of the MTs on a kinesin coated surface formed in the presence of ATP (5 mM). The concentration of the *r*-DNA1 and *r*-DNA2 conjugated MTs, i.e. the red and green MTs 0.6 µM each. The concentration of *l*-DNA1 was 0.6 µM. The concentration of kinesin was 0 in **a,** and 0.3 µM in **b** and **c**. Scale bar: 50 µm.

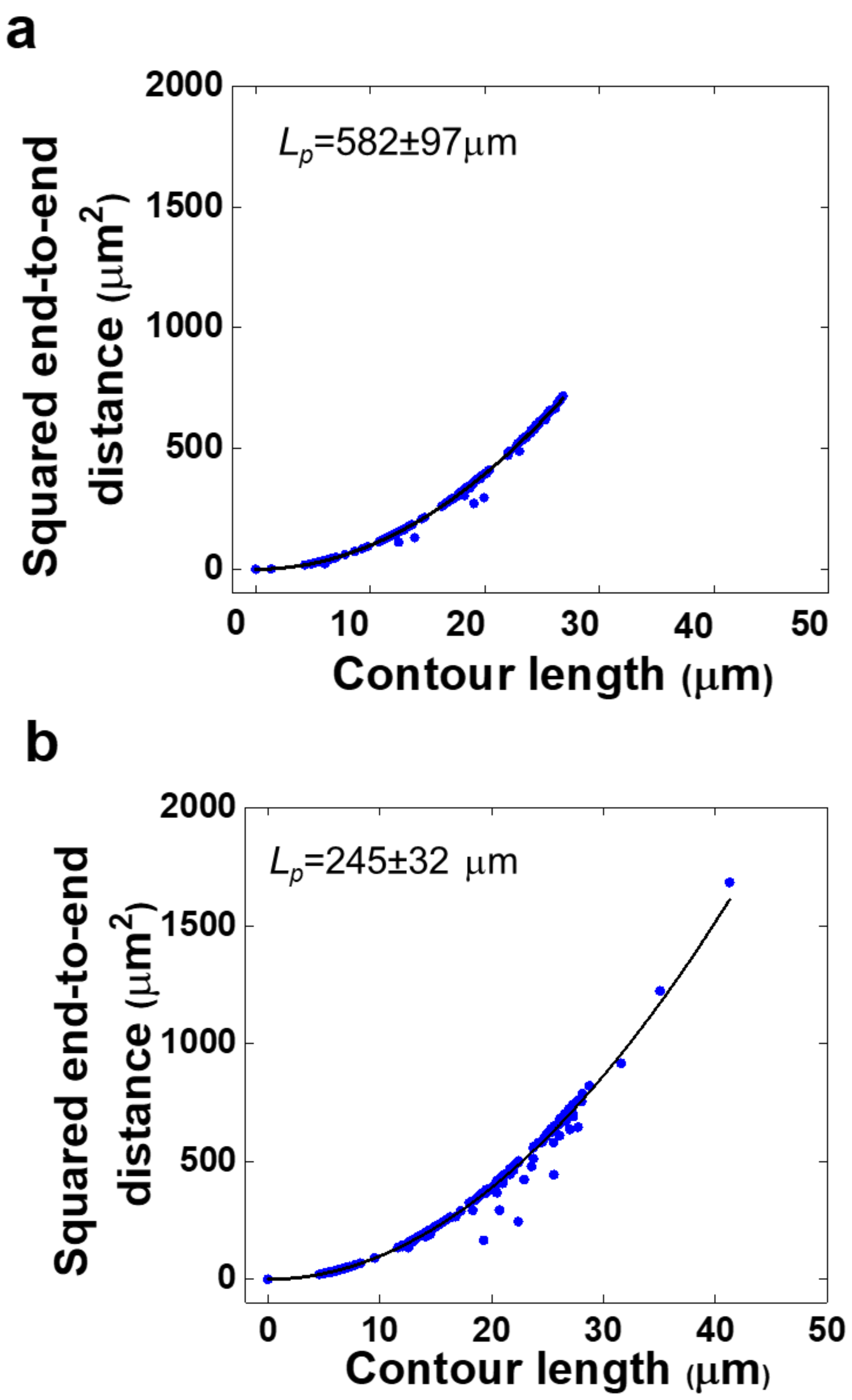


**Supplementary Figure 4. Measurement of the path persistence length ($L_p$) of *r*-DNA1 and *r*-DNA2 conjugated GMPCPP- and GTP-MTs. a,** $L_p$ of rigid MTs (GMPCPP-MTs) and **b,** $L_p$ of flexible MTs (GTP-MTs) from their motility on a kinesin coated substrate. The curves were fitted following the equation, $R^2 = 2L_p{}^2[\frac{L}{L_p} - 1 + \exp(-\frac{L}{L_p})]$, where $R$ is the end to end distance of MTs, $L$ is the contour length of MTs, and $L_p$ is the path persistence length of the MTs. The concentration of kinesin was 0.3 μM.

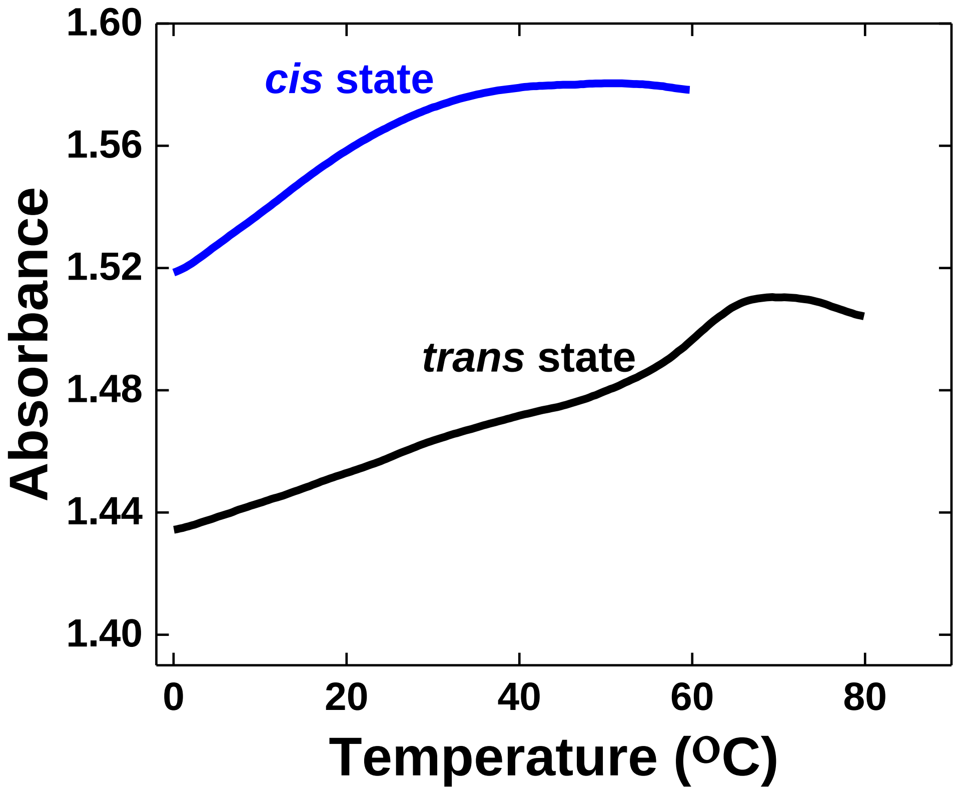


**Supplementary Figure 5. The melting curves of *p*-DNA duplex in the *cis* (blue) and *trans* state (black).** For details of the measurement conditions see Supplementary Methods.

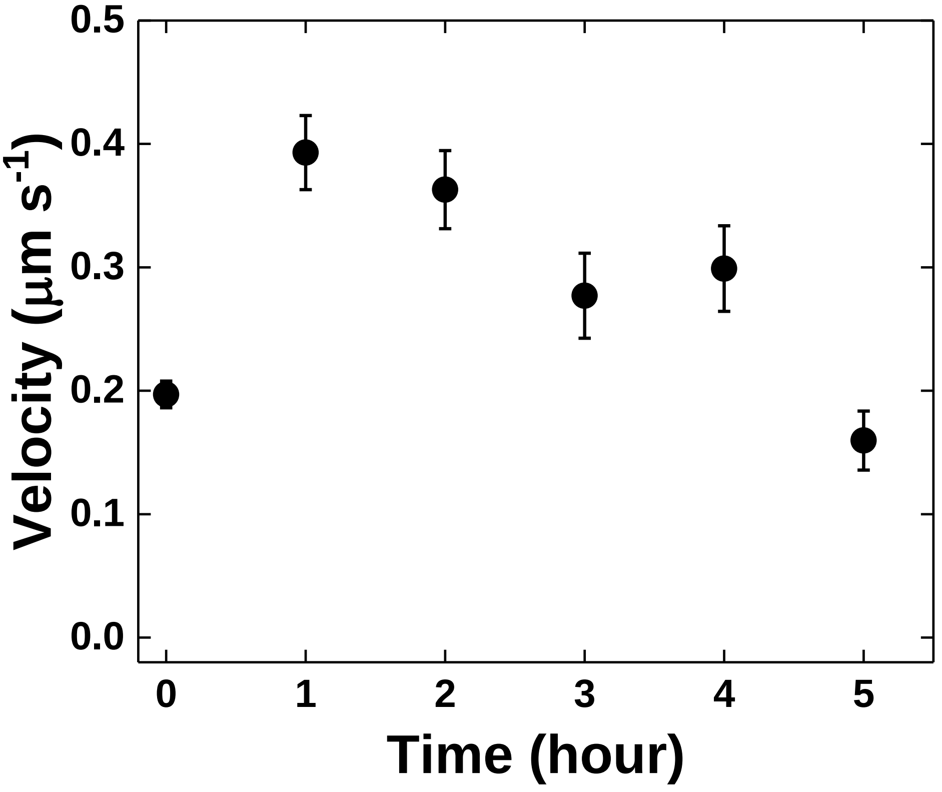


**Supplementary Figure 6. Effect of the conjugation time of *p*-DNA on the velocity of the rigid MTs (GMPCPP-MTs).** The velocity was measured at 25 °C and the number of MTs analyzed was 30. The kinesin concentration was 0.8 μM. Error bar: s.e.m.

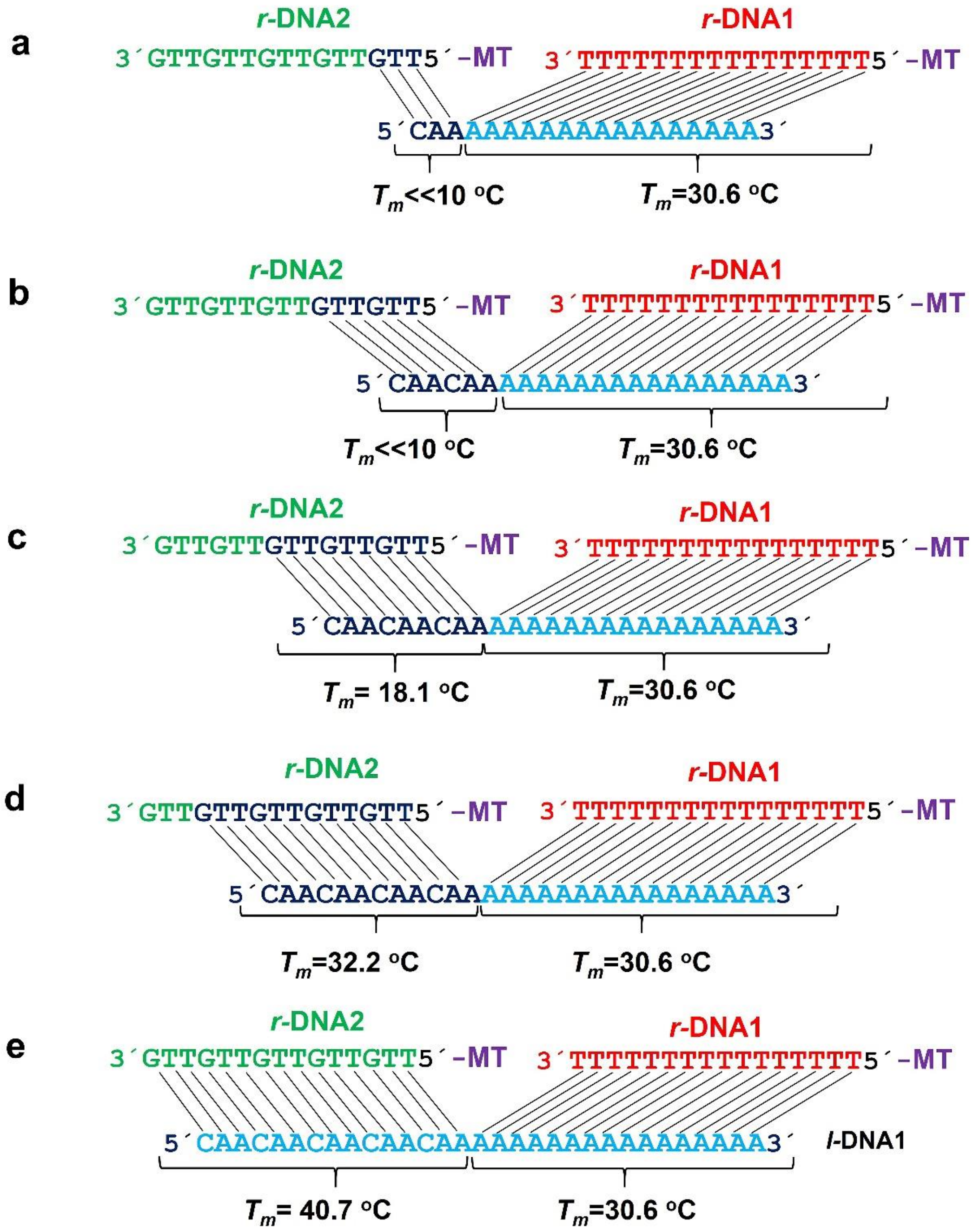

a
r-DNA2
3´GTTGTTGTTGTTGTT5´-MT
r-DNA1
3´TTTTTTTTTTTTTTTTT5´-MT
5´CAAAAAAAAAAAAAAAAAAAA3´
Tm<<10 oC
Tm=30.6 oC
b
r-DNA2
3´GTTGTTGTTGTTGTT5´-MT
r-DNA1
3´TTTTTTTTTTTTTTTTT5´-MT
5´CAACAAAAAAAAAAAAAAAAAAA3´
Tm<<10 oC
Tm=30.6 oC
c
r-DNA2
3´GTTGTTGTTGTTGTT5´-MT
r-DNA1
3´TTTTTTTTTTTTTTTTT5´-MT
5´CAACAACAAAAAAAAAAAAAAAAAAAA3´
Tm= 18.1 oC
Tm=30.6 oC
d
r-DNA2
3´GTTGTTGTTGTTGTT5´-MT
r-DNA1
3´TTTTTTTTTTTTTTTTT5´-MT
5´CAACAACAACAAAAAAAAAAAAAAAAAAA3´
Tm=32.2 oC
Tm=30.6 oC
e
r-DNA2
3´GTTGTTGTTGTTGTT5´-MT
r-DNA1
3´TTTTTTTTTTTTTTTTT5´-MT
5´CAACAACAACAACAAAAAAAAAAAAAAAAAAA3´
l-DNA1
Tm= 40.7 oC
Tm=30.6 oC

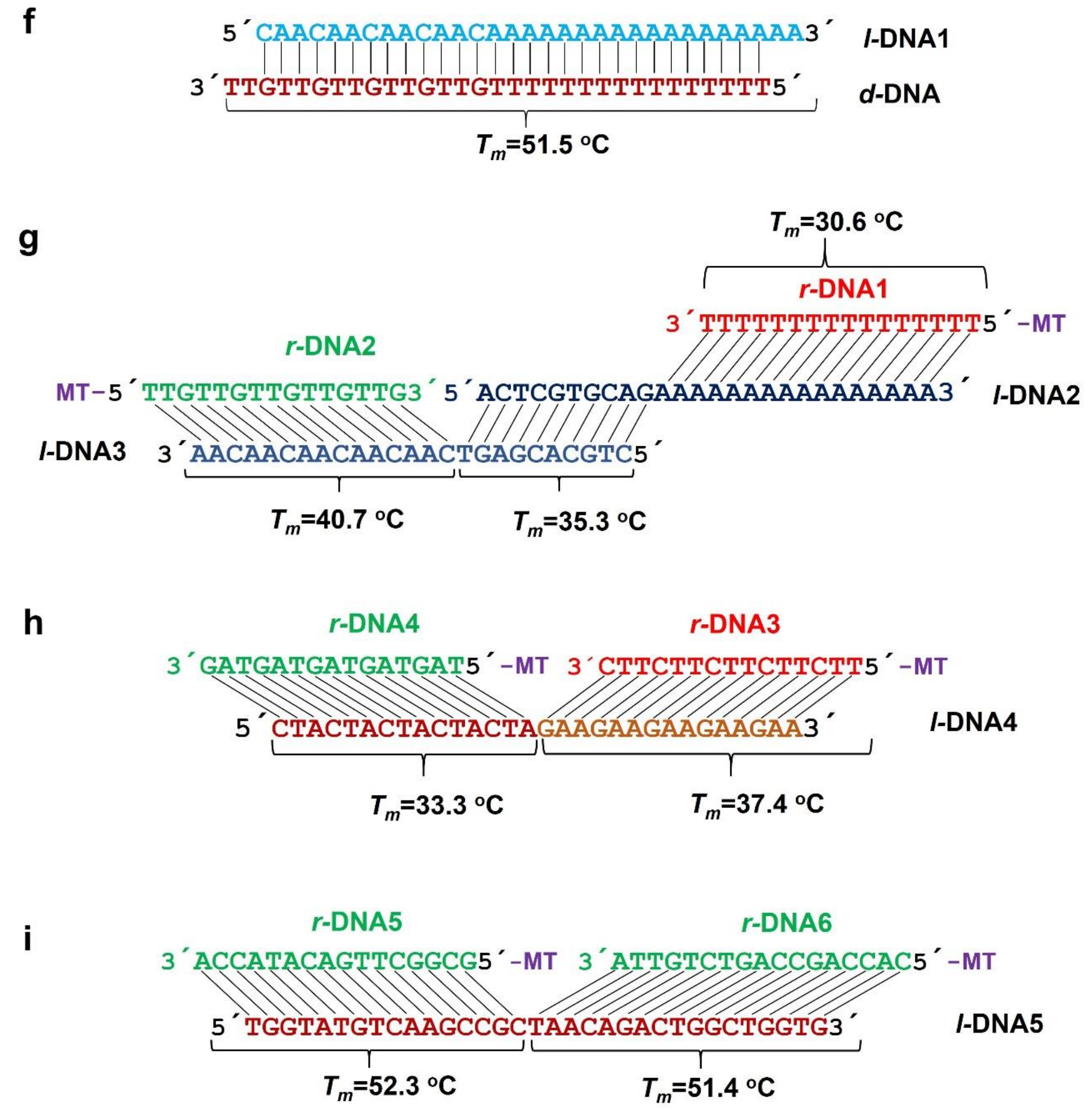


**Supplementary Figure 7. The simulated melting temperature between *r*-DNA and *l*-DNA. (a-i)** Simulation and design of DNA sequences based on $T_m$ to facilitate association and dissociation of swarming of MTs **(a-f)**. **a,** $T_m$ of *r*-DNA1 ($T_{16}$) and *r*-DNA2 ($(TTG)_5$) with $CAAA_{16}$. **b,** $T_m$ of *r*-DNA1 ($T_{16}$) and *r*-DNA2 ($(TTG)_5$) with $(CAA)_2A_{16}$. **c,** $T_m$ of *r*-DNA1 ($T_{16}$) and *r*-DNA2 ($(TTG)_5$) with $(CAA)_3A_{16}$. **d,** $T_m$ of *r*-DNA1 ($T_{16}$) and *r*-DNA2 ($(TTG)_5$) with $(CAA)_4A_{16}$ **e,** $T_m$ of *r*-DNA1 ($T_{16}$) and *r*-DNA2 ($(TTG)_5$) with $(CAA)_5A_{16}$. **f,** $T_m$ of $(CAA)_5A_{16}$ with $T_{16}(GTT)_5$ (used for the DNA strand displacement reaction). **g,** $T_m$ of *r*-DNA1 ($T_{16}$) and *r*-DNA2 ($(TTG)_5$) with *l*-DNA2 ($ACTCGTGCAGA_{16}$) and *l*-DNA3 ($CTGCACGAGT(CAA)_5$) (used in the AND gate operation). **h,** $T_m$ of *r*-DNA3 ($(TTC)_5$) and *r*-DNA4 ($(TAG)_5$) with *l*-DNA5 ($(CTA)_5(GAA)_5$). (used in the OR gate operation with the combination of logic gate using DNA sequences shown in (e)) **i,** $T_m$ for the logic gate of rigid green MTs, *r*-DNA5 (GCGGCTTGACATACCA) and *r*-DNA6 (CACCAGCCAGTCTGTTA) with *l*-DNA5 (TGGTATGTCAAGCCGCTAACAGACTGGCTGGTG) along with the logic gate of flexible red MTs designed with DNA sequences shown in (e). $T_m$ was calculated based on the nearest neighbor method by OligoAnalyzer 3.1.

**Supplementary Table 1.** Labeling ratio of *r*-DNA1 to tubulin dimers.

| *r*-DNA1 concentration in the feed (μM) | Concentration of tubulin dimers (μM) | *r*-DNA1 concentration in tubulin dimers (μM) | Labeling ratio of *r*-DNA1 to tubulin dimers (%) |
|---|---|---|---|
| 10 | 12 | 8 | 70 |
| 100 | 15 | 12 | 84 |
| 300 | 18 | 17 | 93 |
| 500 | 24 | 25 | 101 |
| 1000 | 23 | 25 | 107 |

**Supplementary Table 2.** Labeling ratio of *r*-DNA2 to tubulin dimers.

| *r*-DNA2 concentration in the feed (μM) | Concentration of tubulin dimers (μM) | *r*-DNA2 concentration in tubulin dimers (μM) | Labeling ratio of *r*-DNA2 to tubulin dimers (%) |
|---|---|---|---|
| 10 | 12 | 8 | 66 |
| 100 | 14 | 11 | 76 |
| 300 | 17 | 16 | 92 |
| 500 | 24 | 23 | 96 |
| 1000 | 19 | 21 | 106 |

**Supplementary Table 3.** The sequences of *r*-DNAs, *l*-DNA1, and *d*-DNA used to demonstrate the swarming of MTs.

| **DNA** | **Sequence (5´-3´)** | **5´ end** | **3´ end** |
|---|---|---|---|
| *r*-DNA1 | TTTTTTTTTTTTTTTT | DBCO | TAMRA |
| *r*-DNA2 | TTGTTGTTGTTGTTG | DBCO | FAM |
| *l*-DNA1 | CAACAACAACAACAAAAAAAAAAAAAAAAAA | - | - |
| *d*-DNA | TTTTTTTTTTTTTTTTGTTGTTGTTGTTGTT | - | - |

**Supplementary Table 4.** The sequences of *r*-DNAs and *l*-DNAs used to demonstrate the logic gate operations for the swarming of MTs.

| **DNA** | **Sequence (5´-3´)** | **5´ end** | **3´ end** |
|---|---|---|---|
| *r*-DNA1 | TTTTTTTTTTTTTTTT | DBCO | TAMRA |
| *r*-DNA2 | TTGTTGTTGTTGTTG | DBCO | FAM |
| *l*-DNA1 | CAACAACAACAACAAAAAAAAAAAAAAAAAA | - | - |
| *l*-DNA2 | ACTCGTGCAGAAAAAAAAAAAAAAAA | - | - |
| *l*-DNA3 | CTGCACGAGTCAACAACAACAACAA | - | - |
| *r*-DNA3 | TTCTTCTTCTTCTTC | DBCO | TAMRA |
| *r*-DNA4 | TAGTAGTAGTAGTAG | DBCO | FAM |
| *l*-DNA4 | CTACTACTACTACTAGAAGAAGAAGAAGAA | - | - |

**Supplementary Table 5.** The sequences of *r*-DNAs and *l*-DNAs used to demonstrate the orthogonal control of the swarming of MTs.

| DNA | Sequence (5´-3´) | 5´ end | 3´ end |
|---|---|---|---|
| *r*-DNA1 | TTTTTTTTTTTTTTTT | DBCO | TAMRA |
| *r*-DNA2 | TTGTTGTTGTTGTTG | DBCO | - |
| *l*-DNA1 | CAACAACAACAACAAAAAAAAAAAAAAAAA | - | - |
| *r*-DNA5 | GCGGCTTGACATACCA | DBCO | FAM |
| *r*-DNA6 | CACCAGCCAGTCTGTTA | DBCO | FAM |
| *l*-DNA5 | TGGTATGTCAAGCCGCTAACAGACTGGCTGGTG | - | - |

**Supplementary Table 6.** The sequences of *p*-DNAs used to demonstrate the ON/OFF switching of the swarming of MTs. Z: Azobenzene

| DNA | Sequence (5´-3´) | 5´ end | 3´ end |
|---|---|---|---|
| *p*-DNA1 | CAAZCAAZCAAZCAAZCAAZCAAZCAAZCAA | DBCO | - |
| *p*-DNA2 | TTTTTTTTTTTTTTGZTTGZTTGZTTG | DBCO | - |

## Supplementary References:


1. Asanuma, H. et al. Synthesis of azobenzene-tethered DNA for reversible photo-regulation of DNA functions: hybridization and transcription. *Nat. Protoc.* **2,** 203-212 (2007).
