# Supplementary material for: DNA-assisted swarm control in a biomolecular motor system
Source: Nat Commun. 2018 Jan 31;9:453. doi: 10.1038/s41467-017-02778-5 (PMC5792447; doi:10.1038/s41467-017-02778-5)
Supplement: Supplementary file 3 — Description of Additional Supplementary Files [file 41467_2017_2778_MOESM3_ESM.docx]

**Description of Additional Supplementary Files**

File Name: Supplementary Movie 1

Description: Control of swarming of rigid MTs. Motility of rigid MTs, swarming of rigid MTs with translational motion in the presence of *l*-DNA1 and dissociation of swarms of rigid MTs with *d*-DNA. Scale bar: 20 µm. The movie is 100 times faster than the original speed.

File Name: Supplementary Movie 2

Description: Control of swarming of flexible MTs. Motility of flexible MTs, swarming of flexible MTs with circular motion in the presence of *l*-DNA1 and dissociation of a swarm of flexible MTs with *d*-DNA. Scale bar: 20 µm. The movie is 100 times faster than the original speed.

File Name: Supplementary Movie 3

Description: Orthogonally controlled swarming of MTs with translational and circular motion. Scale bar: 20 µm. The movie is 100 times faster than the original speed.

File Name: Supplementary Movie 4

Description: Control of on/off switching of swarming of MTs by photoirradiation. Control of repeated reversible swarming of rigid MTs (right) and flexible MTs (left) by UV and visible light irradiation. Scale bar: 20 µm. The movie is 100 times faster than the original speed.
